# Advancing IoT in the lab: Next generation Gateway-Module for laboratory device integration

**DOI:** 10.1016/j.ohx.2025.e00688

**Published:** 2025-09-06

**Authors:** Ferdinand Lange, Sascha Beutel

**Affiliations:** Leibniz University Hannover, Institute of Technical Chemistry, Callinstraße 5, 30167 Hannover, Germany

**Keywords:** Digital integration, Embedded computing, PCB, Internet of Things (IoT), Laboratory, Digitization

## Abstract

This article introduces the next generation of the Gateway-Module, which is designed to simplify experimental setups and to further advance digitization. Based on the innovative work of Porr et al. in 2020, the Gateway-Module-v3 replaces the single board computer (SBC) with a Phytec i.MX 8M Plus (phycore-imx8mp) System on Module (SoM). This upgrade significantly reduces the physical size of the module to a compact 5.4 cm width, length, and height. Gateway-Module-v3 seamlessly integrates with standards such as Standard in Laboratory Automation (SiLA 2) and Open Platform Communication Unified Architecture (OPC-UA), advancing the digitization and efficiency of laboratory operations. At the center of its innovation is the tty2eth management module, which provides robust remote monitoring and maintenance using SSH and Prometheus metrics to ensure that experiments run smoothly even from remote locations. The Gateway-Module-v3 offers a wide range of connectivity options, including GPIO, USB and serial ports, making it adaptable to a variety of applications. Rigorous stress testing has confirmed its reliability and performance under demanding conditions, highlighting the suitability for both laboratory and remote scenarios. With its advanced functionality and connectivity, the Gateway-Module-v3 is ready to support researchers, advance their work, and will serve as a fundamental resource for current and future needs.

## Graphical abstract

**Figure:**
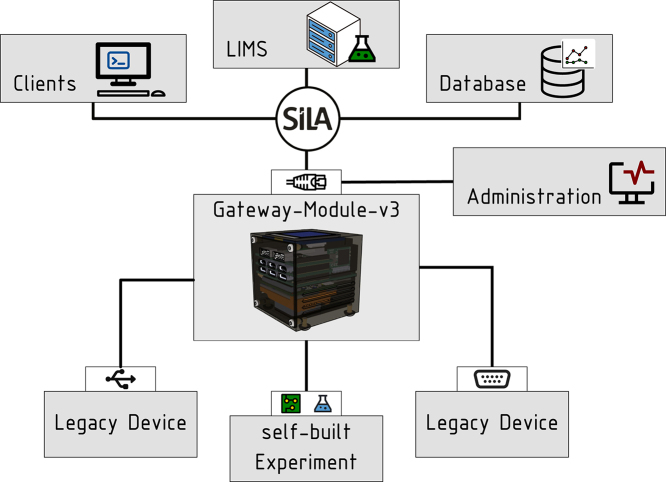

**Specifications table**

**Table:** 

Hardware name	Gateway-Module-v3
Subject area	•Chemistry, Biochemistry and Biotechnology • Research Laboratories • Educational Tools and Open Source Alternatives to Existing Infrastructure • System Integration, Digitization and Digitalization
Hardware type	•System Integration Gateway • Electrical engineering and computer science
Closest commercial analog	LabOperator form Labforward
Open source license	MIT-License: Copyright (c) 2025 Institut f‘`ur Technische Chemie, Leibniz Universit’´at Hannover Permission is hereby granted, free of charge, to any person obtaining a copy of this hardware, software, and associated documentation files (the “Product”), to deal in the Product without restriction, including without limitation the rights to use, copy, modify, merge, publish, distribute, sublicense, and/or sell copies of the Product, and to permit persons to whom the Product is furnished to do so, subject to the following conditions: The above copyright notice and this permission notice shall be included in all copies or substantial portions of the Product. THE PRODUCT IS PROVIDED “AS IS”, WITHOUT WARRANTY OF ANY KIND, EXPRESS OR IMPLIED, INCLUDING BUT NOT LIMITED TO THE WARRANTIES OF MERCHANTABILITY, FITNESS FOR A PARTICULAR PURPOSE AND NONINFRINGEMENT. IN NO EVENT SHALL THE AUTHORS OR COPYRIGHT HOLDERS BE LIABLE FOR ANY CLAIM, DAMAGES OR OTHER LIABILITY, WHETHER IN AN ACTION OF CONTRACT, TORT OR OTHERWISE, ARISING FROM, OUT OF OR IN CONNECTION WITH THE PRODUCT OR THE USE OR OTHER DEALINGS IN THE PRODUCT.
Cost of hardware	650 Euros
Source file repository	https://doi.org/10.17632/p6vvtyvbsb.1

## 1 Hardware in context

This article presents a detailed description of the improved and minimized Gateway-Module-v3. The previous version was developed by Marc Porr et al. [1]. Since its publication the demand for computer controlled experiments and digitalization in laboratories has continued to rise [2], [3], [4], [5]. This is for various reasons, such as digitizing legacy laboratory equipment, executing self-built computer-controlled experiments, and using SBC to support sensor development. In these areas a mini PC that functions as a mini server has advantages over Windows PCs or laptops, including requiring significantly less space. During server operation, the application can be monitored and controlled from the network on different remote devices. Laboratory device communication standards, such as SiLA 2 or OPC-UA, can be used for this purpose [6], [7], [8], [9]. The use of web applications or REST services is also widespread [10].

The new Gateway-Module-v3 is primarily intended to assist in the development of laboratory equipment and self-built experimental setups, such as those described in [11], [12]. It has been equipped with a new module to facilitate hardware monitoring and maintenance. Moreover, the dimensions of the Gateway-Module-v3 have been reduced to 5.4 cm in length, width, and height. Modularity has increased by switching from the SBC to a SoM, allowing the hardware to be easily updated if necessary.

While developing the focus was on laboratory digitization. A variety of connection options allow for integration with laboratory devices and experimental setups, enabling device control and data processing in accordance with Findability, Accessibility, Interoperability, and Reusability (FAIR) criteria [13], [14], [15]. Along with its uses in laboratories, the new Gateway-Module-v3 can also be used in other areas where SBCs are useful. Additionally, extra maintenance functionalities enable operations at remote locations.

## 2 Hardware description

The goal of this project was to create an enhanced version of the previously developed Gateway-Module [1] with the following improvements:

Size: Reduce the physical size of the Gateway-Module on the laboratory bench or inside other devices and reduce the necessary wiring.
Remote maintenance: Add the ability to remotely control the Gateway-Module at the hardware level and monitor status information of this device.
Hardware Platform: Provide a hardware platform for self-build devices and experiments.
Modularity: Enables flexible connectivity.

**Fig. 1:**
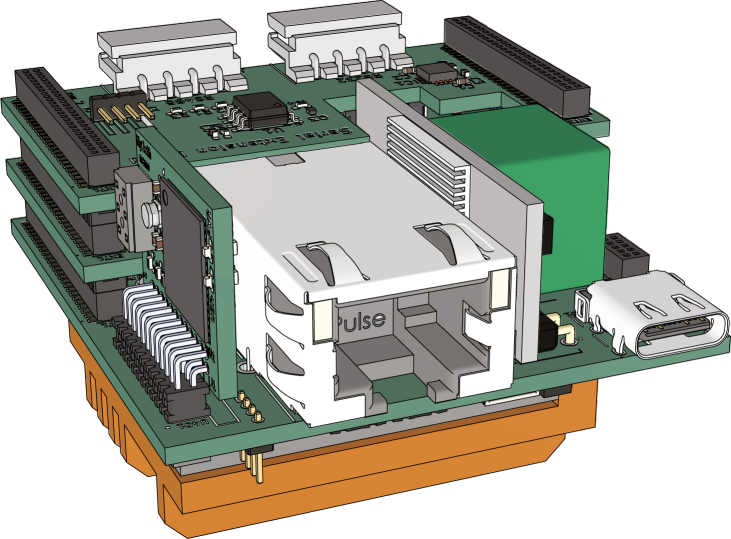
The Gateway-Module-v3.

The result of this work is the Gateway-Module-v3 as shown in Fig. 1.

### 2.1 Architectural overview

**Fig. 2:**
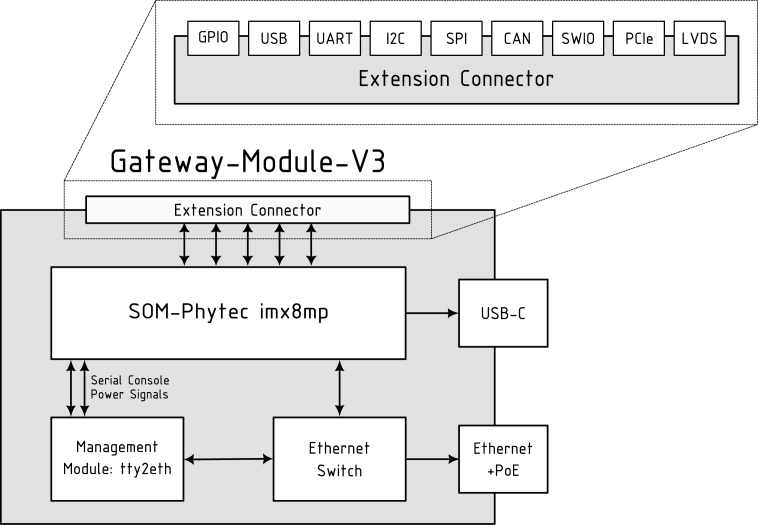
An overview of the Gateway-Module-v3 architecture, with the main components: the phycore-imx8mp SoM, the integrated Ethernet switch, the tty2eth management module, and the expansion ports.

This version of the Gateway-Module contains the following main components as shown in Fig. 2 and described in more detail in the following sections.

#### 2.1.1 The phycore-imx8mp SoM

The Gateway-Module-v3 is constructed from multiple components, with its core element being the phycore-imx8mp a SoM. It was a primary objective to minimize its physical dimensions. Therefore, it has been determined that the SBC, Odroid-C2 [16] of the original Gateway-Module and the Odroid-C4 [17] of the internal Gateway-Module-v2 have been substituted with a SoM. The transition from a SBC to a SoM was advantageous for a number of reasons. These include the SoM’s compact dimensions and its modularity, both of which align with the Gateway-Module-v3 requirements. This architecture provides a robust foundation for the Gateway-Module-v3 requirements and facilitates a wide range of connectivity options.

In order to maintain a high degree of flexibility in the selection of necessary performance characteristics and to ensure that the dimensions of the new Gateway-Module-v3 remain minimal, the phycore-imx8mp with dimensions of 40mm×37mm was the SoM of choice for this particular version. The device’s architecture is supported by an ARM Cortex-A53 processor, a LPDDR4 RAM module, and an embedded MultiMedia Card (eMMC) data storage unit. A salient benefit of utilizing a SoM over a SBC is the flexibility to adjust the computing power as required by substituting the module with a more advanced one. Phytec, the manufacturer, provides the phycore-imx8mp in a variety of performance specifications that are interchangeable [18].

#### 2.1.2 tty2eth management module

In the course of developing and improving laboratory equipment [19], the challenge of having the Gateway-Module permanently installed in the laboratory while the software was being developed in the office was repeatedly encountered. This complication made it difficult to debug the software at hardware level, particularly during the boot sequence when remote connections were inaccessible.

To improve the development and management of these devices, inspiration was drawn from the server industry. The integration of small mini-PCs, known as Baseboard Management Controllers (bmcs), within servers enables remote maintenance and monitoring of these systems [20], [21].

**Fig. 3:**
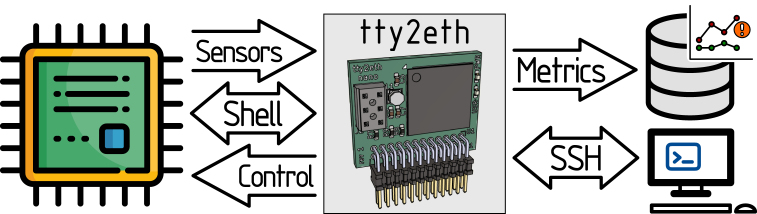
This is an overview of the tty2eth module’s functionalities.

As illustrated in Fig. 3, the tty2eth module’s functionality includes the monitoring and processing the serial console of the phycore-imx8mp and the sensor values of the Gateway-Module-v3, which include temperature and power consumption. Additionally, the transmission of start and stop signals and commands to the SoM is possible. These functions can be accessed via a secure connection using the Secure Shell (SSH) protocol. Moreover, the monitoring metrics of the sensors are provided as Prometheus metrics [22], [23]. These functionalities enable the monitoring and visualization of the health status of one or more Gateway-Module-v3 [24], [25]. The implementation of alarms can be achieved through the utilization of Prometheus or Grafana [26]. In the event of a critical sensor value, the user is notified and can take action.

#### 2.1.3 Integrated ethernet switch

The integrated Ethernet switch establishes a connection between the SoM and the tty2eth module and also enabling external communication via an Ethernet interface. The Ethernet port also allows the Gateway-Module-v3 to be powered via Power over Ethernet (PoE). This means that the Gateway-Module-v3 can be used with an ethernet cable only.

#### 2.1.4 Extensions

The aim of the Gateway-Module-v3 is to be used in as many areas as possible, from operating various legacy devices to controlling self-build sensors and complex experimental setups. To achieve this, the Gateway-Module-v3 offers a wide range of connectivity and expansion options:

• 13×General Purpose Input/Output (GPIO)
• 1×Universal Serial Bus (USB)
• 3×Universal Asynchronous Receiver-Transmitter (UART)
• 3×Inter-Integrated Circuit (I2C)
• 2×Serial Peripheral Interface (SPI)
• 2×Secure Digital Input/Output (SDIO)
• 1×Peripheral Component Interconnect Express (PCIe)
• 1×Controller Area Network (CAN)
• 1×Low-Voltage Differential Signaling (LVDS)

For quick start, the project provides two ready-made extension boards, one for USB and one for serial connections. In addition, there is a KiCad template to support the development of further extension boards to extend the functionality of Gateway-Module-v3 as desired.

##### Serial extension board:.

The serial extension board offers one RS232 (UART2) and one RS485 (UART3) connection in the form of a Molex 22-05-7045 connector. A conventional D-Sub 9 connector was not used for space reasons. In addition, the board offers an internal connection option for a I2C device, such as a simple OLED display.

##### USB extension board:.

The Gateway-Module-v3 itself only offers one USB 3.0 port. However, this port can be expanded to 6 USB-C 2.0 ports using the USB extension board with its integrated USB hub. In addition, this extension board offers another USB 3.0 port on the upper side of the extension connectors. This means that any number of USB extension boards can be connected to each other. However, the power supply of the USB devices must be taken into account here, as the Gateway-Module-v3 cannot supply an unlimited amount of power.

### 2.2 Comparison to other solutions

The Gateway-Module-v3 offers a wide range of connectivity options through various bus and connector standards, some of which are only available on other SBCs with separate boards, such as the PiCAN2 [27], which provides a CAN bus over SPI for Raspberry Pi. Compared to the original Gateway-Module, version 3 is significantly smaller with an edge length of 5.4 cm and can be used in a variety of ways through its modularity [1]. The integrated management module, tty2eth, simplifies development compared to conventional SBCs, such as the Raspberry Pi or the Odroid-C4. This is particularly useful for devices that are permanently installed and not always available for debugging.

### 2.3 Potential use to other researchers

The development of the Gateway-Module-v3 was primarily centered on the areas of laboratory digitization and the controlling of self-build experiments. However, it is noteworthy that the scope of the Gateway-Module-v3 extends beyond the laboratory, and it may find applications in other fields, including but not limited to:

• The use of SBCs in remote and hard-to-reach applications
• The firmware of the tty2eth module can be extended so that the main system is started only for certain events and performs the necessary computations or data processing before shutting down after successful completion to save power
• For hobbyists who want to solve complex tasks with a modular platform

## 3 Design files summary

**Table:** 

**Design filename**	**File type**	**Open source license**	**Location of the file**
tty2eth-nano.pdf	schematics	MIT	pcbs/schematics/tty2eth-nano.pdf
gwm-main-board.pdf	schematics	MIT	pcbs/schematics/gwm-main-board.pdf
serial-extension.pdf	schematics	MIT	pcbs/schematics/serial-extension.pdf
usb-extension.pdf	schematics	MIT	pcbs/schematics/usb-extension.pdf
serial-extension.html	Interactive BOM	MIT	pcbs/bom/serial-extension.html
tty2eth-nano.html	Interactive BOM	MIT	pcbs/bom/tty2eth-nano.html
gwm-main-board.html	Interactive BOM	MIT	pcbs/bom/gwm-main-board.html
usb-extension.html	Interactive BOM	MIT	pcbs/bom/usb-extension.html
usb-extension_1.zip	Production Gerber files	MIT	pcbs/production/usb-extension_1.zip
tty2eth-nano_1.zip	Production Gerber files	MIT	pcbs/production/tty2eth-nano_1.zip
serial-extension_2.zip	Production Gerber files	MIT	pcbs/production/serial-extension_2.zip
gwm-main-board_1.zip	Production Gerber files	MIT	pcbs/production/gwm-main-board_1.zip
case-base.step	3D case step files	MIT	3d-case/case-base.step
foot.step	3D case step files	MIT	3d-case/foot.step
fan-grill.step	3D case step files	MIT	3d-case/fan-grill.step
backplate.step	3D case step files	MIT	3d-case/backplate.step
gateway-module-v3-case.step	3D step files	MIT	3d/gateway-module-v3-case.step
gateway-module-v3.step	3D step files	MIT	3d/gateway-module-v3.step
tty2eth-nano.step	3D step files	MIT	3d/tty2eth-nano.step
XK049b-Heatspreader.step	3D step files	phytec	3d/XK049b-Heatspreader.step
fan_25 × 25 × 6.step	3D step files	MIT	3d/fan_25 × 25 × 6.step
gateway-module-v3-full.step	3D step files	MIT	3d/gateway-module-v3-full.step
usb-extension.step	3D step files	MIT	3d/usb-extension.step
serial-extension.step	3D step files	MIT	3d/serial-extension.step
phytec-imx8mp.step	3D step files	phytec	3d/phytec-imx8mp.step
openocd.cfg	firmware	MIT	firmware/openocd.cfg
alpine-box-gwm-v3.img.zst	firmware	MIT	firmware/alpine-box-gwm-v3.img.zst
tty2eth.elf	firmware	MIT	firmware/tty2eth.elf
flash.bin	firmware	MIT	firmware/flash.bin
demo.py	Showcase project files	MIT	demo/demo.py
Ismatec30.py	Showcase project files	MIT	demo/Ismatec30.py
OLED_1in5.py	Showcase project files	MIT	demo/OLED_1in5.py
demo-2025-05-08T19_43_47.281769.json	Showcase project files	MIT	demo/demo-2025-05-08T19_43_47.281769.json
demo-gwm-v3.schema.json	Showcase project files	MIT	demo/demo-gwm-v3.schema.json
gwm-main-board.kicad_pro	kicad project file	MIT	pcbs/gwm-main-board/gwm-main-board.kicad_pro
phytec.kicad_sch	schematics	MIT	pcbs/gwm-main-board/phytec.kicad_sch
phytec_CD.kicad_sch	schematics	MIT	pcbs/gwm-main-board/phytec_CD.kicad_sch
usb-c.kicad_sch	schematics	MIT	pcbs/gwm-main-board/usb-c.kicad_sch
stm32.kicad_sch	schematics	MIT	pcbs/gwm-main-board/stm32.kicad_sch
gwm-main-board.kicad_sch	schematics	MIT	pcbs/gwm-main-board/gwm-main-board.kicad_sch
tty2eth.kicad_sch	schematics	MIT	pcbs/gwm-main-board/tty2eth.kicad_sch
extension.kicad_sch	schematics	MIT	pcbs/gwm-main-board/extension.kicad_sch
switch.kicad_sch	schematics	MIT	pcbs/gwm-main-board/switch.kicad_sch
phytec_AB.kicad_sch	schematics	MIT	pcbs/gwm-main-board/phytec_AB.kicad_sch
power.kicad_sch	schematics	MIT	pcbs/gwm-main-board/power.kicad_sch
gwm-main-board.kicad_pcb	board design file	MIT	pcbs/gwm-main-board/gwm-main-board.kicad_pcb
tty2eth-nano.kicad_pro	kicad project file	MIT	pcbs/tty2eth-nano/tty2eth-nano.kicad_pro
sensors.kicad_sch	schematics	MIT	pcbs/tty2eth-nano/sensors.kicad_sch
stm32.kicad_sch	schematics	MIT	pcbs/tty2eth-nano/stm32.kicad_sch
tty2eth-nano.kicad_sch	schematics	MIT	pcbs/tty2eth-nano/tty2eth-nano.kicad_sch
phy.kicad_sch	schematics	MIT	pcbs/tty2eth-nano/phy.kicad_sch
tty2eth-nano.kicad_pcb	board design file	MIT	pcbs/tty2eth-nano/tty2eth-nano.kicad_pcb
extension-template.kicad_pro	kicad project file	MIT	pcbs/extensions/extension-template/extension-template.kicad_pro
connector.kicad_sch	schematics	MIT	pcbs/extensions/extension-template/connector.kicad_sch
extension-template.kicad_sch	schematics	MIT	pcbs/extensions/extension-template/extension-template.kicad_sch
extension-template.kicad_pcb	board design file	MIT	pcbs/extensions/extension-template/extension-template.kicad_pcb
serial-extension.kicad_pro	kicad project file	MIT	pcbs/extensions/serial-extension/serial-extension.kicad_pro
connector.kicad_sch	schematics	MIT	pcbs/extensions/serial-extension/connector.kicad_sch
serial.kicad_sch	schematics	MIT	pcbs/extensions/serial-extension/serial.kicad_sch
i2c.kicad_sch	schematics	MIT	pcbs/extensions/serial-extension/i2c.kicad_sch
serial-extension.kicad_sch	schematics	MIT	pcbs/extensions/serial-extension/serial-extension.kicad_sch
serial-extension.kicad_pcb	board design file	MIT	pcbs/extensions/serial-extension/serial-extension.kicad_pcb
usb-extension.kicad_pro	kicad project file	MIT	pcbs/extensions/usb-extension/usb-extension.kicad_pro
connector.kicad_sch	schematics	MIT	pcbs/extensions/usb-extension/connector.kicad_sch
usb-connectors.kicad_sch	schematics	MIT	pcbs/extensions/usb-extension/usb-connectors.kicad_sch
usb-hub.kicad_sch	schematics	MIT	pcbs/extensions/usb-extension/usb-hub.kicad_sch
usb-extension.kicad_sch	schematics	MIT	pcbs/extensions/usb-extension/usb-extension.kicad_sch
usb-extension.kicad_pcb	board design file	MIT	pcbs/extensions/usb-extension/usb-extension.kicad_pcb

### 3.1 Design file descriptions

This project contains the following design files and can be categorized into:

pcbs/**: Contains all necessary files for the different Kicad projects and the included Printed Circuit Board (PCB) design files. The PCBs are separated in: **gwm-main-board**, **tty2eth-nano**, **serial-extension** and **usb-extension**.
*.kicad_pro: the Kicad project files of the PCB
*.kicad_sch: the Kicad schematic file definition for the PCB project
*.kicad_pcb: the Kicad board design files for the PCB project
pcbs/schematics/*.pdf: Schematics as pdf of the different PCBs
pcbs/bom/*.csv: Comma Separated Values (CSV) based Bill of Materials (BOM) file of the PCB
pcbs/bom/*.html: Interactive BOM file as guild line for the PCB assembly
pcbs/production/*.zip: Zip files containing all necessary Gerber files for production at JLCPCB
3d-case/*.step: Contains all 3D model files for printing the Gateway-Module-v3 case
3d/*.step: 3D models of the separate component, can be used to create a different backplate for the case
tty2eth.elf: The precompiled firmware of the tty2eth module
openocd.cfg: The openocd configuration used to flash the firmware to the tty2eth module using a stlink.
alpine-box-gwm-v3.img.zst: Is a compressed base linux image for the Gateway-Module-v3
flush.bin: is the U-Boot bootloader of the phycore-imx8mp SoM
demo/*.py: Python script files of the showcase
demo/*.json: JSON data file of the showcase
demo/demo-gwm-v3.schema.json: JSON schema for the data from the showcase

The files **XK049b-Heatspreader.step** and **phytec-imx8mp.step** were downloaded from the Phytec homepage [28].

### 3.2 Source code repositories

The main Repository [29] contains only a prebuild version of the firmware for the tty2eth module and a prebuild version of the Linux system. The source code of the tty2eth firmware can be found at [30]. This Linux image was build with the mkbox-project [31] and the repository can be found [32].

## 4 Bill of materials summary

This section lists the materials necessary to construct a copy of the Gateway-Module-v3. It should be noted that all prices are exclusive of shipping fees and VAT. Table 1 provides a general overview of the necessary components. The detailed parts lists and links for each of the PCBs are listed in Sections 4.1–4.4.

**Table 1:** The overall bill of materials for the Gateway-Module-v3.

**Designator**	**Component**	**Number**	**Cost per unit EUR**	**Total cost EUR**	**Source of materials**
gateway-module-v3	gateway-module-v3 BOM	1	112.39	112.39	Mouser
gateway-module-v3	gateway-module-v3 PCB	1	75.56	75.56	jlcpcb
tty2eth	tty2eth BOM	1	22.50	22.5	Mouser
tty2eth	tty2eth PCB	1	6.15	6.15	jlcpcb
usb-extension	usb-extension BOM	1	51.60	51.6	Mouser
usb-extension	usb-extension PCB	1	26.36	26.36	jlcpcb
serial-extension	serial-extension BOM	1	40.47	40.47	Mouser
serial-extension	serial-extension PCB	1	6.15	6.15	jlcpcb
STLINK-V3SET	STLINK-V3SET	1	34.58	34.58	STMicroelectronics
phytec-imx-8mp	phytec-imx-8mp	1	154.00	154	phytec
Fan 25 × 25 × 6	MF25060V21000UA99	1	8.67	8.67	Sunon
OLED Screen	1.5inch OLED Module (B)	1	10.55	10.55	Waveshare
Molex Pi-coBlade 4 Pin Housings	51021-0400	2	0.18	0.36	Molex
Molex Pi-coBlade Pins	50079-8001	8	0.10	0.8	Molex
Heatsink	XK049b	1	36.48	36.48	PCBWAY
Heat Pad	40015020	1	13.59	13.59	Wurth Elektronik
M2 × 4 × 3.2 Threaded Insert	MSIB-M2-400	8	0.22	1.76	CI
M2 × 5 Screw	91292A005	8	0.13	1.04	mcmaster
M2 × 10 Screw	91292A833	4	0.06	0.24	mcmaster
M2.5 × 15 Screw	92125A085	2	0.07	0.14	mcmaster
M2.5 Nut	91828A113	2	0.06	0.12	mcmaster
Base Case	3D Case	1	15.69	15.69	PCBWAY
Foot Case	3D Case	4	4.37	17.48	PCBWAY
Fan Grill	3D Case	1	10.93	10.93	PCBWAY
Backplate	3D Case	1	10.93	10.93	PCBWAY

			Overall cost:	658.50	

### 4.1 Gateway-module-v3 — BOM

**Table:** 

**Designator**	**Component**	**Number**	**Cost per unit EUR**	**Total cost EUR**	**Source of materials**
BR501-BR502	652-CD-HD01	2	0.59	1.18	Bourns
C201-C208, C301-C307, C310-C320, C323-C334, C507, C601-C605	81-GRM033Z71C104KE4J	44	0.09	3.96	Murata
C308-C309, C321-C322	963-MEASA105CB5226NA	4	0.19	0.76	Taiyo Yuden
C502	661-APSE6R3L471MF08S	1	0.41	0.41	United Chemi-Con
C504, C506	187-CL05A106MP6NUN8	2	0.11	0.22	Samsung Electro-Mechanics
C505, C801	187-CL05A105KO5NNNC	2	0.09	0.18	Samsung Electro-Mechanics
Cff501	581-06035A100J	1	0.09	0.09	KYOCERA AVX
Cin501	810-C2012X5R1V226MAC	1	0.52	0.52	TDK
Cout501	81-GRM32ER71A476KE5L	1	0.89	0.89	Murata
Css501	80-C0603X472J4RECAUT	1	0.15	0.15	KEMET
D1-D4, D303	750-CDBQR40	5	0.24	1.2	Comchip
D301, D801	755-SML-P11MTT86R	2	0.34	0.68	ROHM
D302, D802	755-SML-P11YTT86R	2	0.51	1.02	ROHM
D501	576-SMAJ58A	1	0.26	0.26	Littelfuse
D502-D503	755-RB080AR-40T7R	2	0.79	1.58	ROHM
D601-D603	595-TPD4E02B04DQAR	3	0.58	1.74	Texas Instruments
EXT1-EXT2	571-1MMRD25VS00FTBP	2	7.33	14.66	TE Connectivity / AMP
FB301-FB303	810-MPZ1005S121HT000	3	0.10	0.3	TDK
H801	534-3030TR	1	0.42	0.42	Keystone
IC501	PEM1405	1	18.31	18.31	INFOMART IT SOLUTIONS
IC503	595-TPSM82810SILR	1	3.73	3.73	Texas Instruments
IC504	511-LD57100J120R	1	0.48	0.48	
IC505	595-INA4235AIYBJR	1	6.86	6.86	Texas Instruments
IC601	595-HD3SS3220IRNHT	1	3.37	3.37	Texas Instruments
IC602	595-TPS66021YBGR	1	0.92	0.92	Texas Instruments
J301	673-JT41202HL	1	9.06	9.06	Pulse Electronics
J601	640-USB4056-03-A	1	0.87	0.87	GCT
J901	200-TMS10402FS	1	0.74	0.74	Samtec
J902	571-1MMRD05VS00FTBP	1	2.10	2.1	TE Connectivity / AMP
PS501	595-TPS22976DPUR	1	0.51	0.51	Texas Instruments
Q501, Q801-Q802	621-DMN1260UFA-7B	3	0.29	0.87	Diodes Inc.
R301-R302, R304, R505, R806	652-CR0402FX-4701GLF	5	0.09	0.45	Bourns
R303, R306, R328	652-CR0402AFX1001GLF	3	0.09	0.27	Bourns
R311-R312, R315-R318, R321	652-CR0402FX-33R0GLF	7	0.09	0.63	Bourns
R322	667-ERJ-2RKF6041X	1	0.09	0.09	Panasonic
R323-R326, R801-R802	652-CR0402FX-2700GLF	6	0.09	0.54	Bourns
R501-R502, R508	708-CSS0603FT2L00	3	0.34	1.02	Stackpole Electronics1
R506	CR0402-FX-1212GLF	1	0.02	0.02	Bourns
R509-R510, R807, Rcf501	71-CRCW0402-10K-E3	4	0.11	0.44	Vishay
R601	603-AC0402FR-07909KL	1	0.09	0.09	Yageo
R602-R607	652-CR0402FX-2003GLF	6	0.09	0.54	Bourns
R608	652-CR0402FX-7502GLF	1	0.09	0.09	Bourns
R609	652-CR0402-FX1023GLF	1	0.09	0.09	Bourns
R610	652-CR0402FX-2002GLF	1	0.09	0.09	Bourns
R803-R804	652-CR0201-J/-000GLF	2	0.09	0.18	Bourns
R805, Rfbb501, Rpg501	71-CRCW0402-100K-E3	3	0.11	0.33	Vishay
Rfbt501	71-CRCW0402-453K-E3	1	0.09	0.09	Vishay
S1-S2	179-TS204225WT160SMT	2	0.26	0.52	Same Sky
SW801	229-CVS-04TB	1	3.01	3.01	Nidec Co-pal1111
U301	579-KSZ9563RNXC	1	8.36	8.36	Microchip
U401	200-CLP11202FDBE	1	3.26	3.26	Samtec1
X201a,X201b	200-BTH06001LDAKTR	2	4.47	8.94	Samtec
X201c	200-BTH03001LDAKTR	1	3.93	3.93	Samtec
Y301	520-1612MV-250-CNT	1	1.46	1.46	ECS International

			Overall cost:	112.39	

Note on component X201 from schematics and board files: it is the connector to the SoM and contains three separate parts X201a - X201c as listed in this BOM.

### 4.2 tty2eth addon

**Table:** 

**Designator**	**Component**	**Number**	**Cost per unit EUR**	**Total cost EUR**	**Source of materials**
C1, C6-C7	187-CL05A106MP6NUN8	3	0.11	0.33	Samsung Electro-Mechanics
C2-C3, C12	187-CL05A475MP5NRNC	3	0.09	0.27	Samsung Electro-Mechanics
C4, C8, C11, C13-C20, C23-C25	80-C0402C104K4R7867	14	0.11	1.54	KEMET
C5	187-CL05C221JB5NNNC	1	0.09	0.09	Samsung Electro-Mechanics
C9-C10	187-CL05A105KO5NNNC	2	0.09	0.18	Samsung Electro-Mechanics
C21-C22	963-MSASL105SB5225KF	2	0.09	0.18	Taiyo Yuden
FB1	81-BLM15PE300SH1D	1	0.21	0.21	Murata
IC1	700-MAX31875R2TZS+T	1	1.42	1.42	Analog Devices
J1	200-FTSH11204FDRA	1	2.66	2.66	Samtec
J2	710-62000621121	1	0.30	0.3	Wurth Elektronik
L1	963-MDMK2020T2R2MM	1	0.26	0.26	Taiyo Yuden
LED_PWR1	755-SML-P11MTT86R	1	0.34	0.34	ROHM
LED_USR1	755-SML-P11UTT86R	1	0.37	0.37	ROHM
R1-R2	652-CR0402AFX1001GLF	2	0.09	0.18	Bourns
R3	652-CR0402AFX1002GAS	1	0.09	0.09	Bourns
R4	652-CR0402FX-47R0GLF	1	0.09	0.09	Bourns
R5-R6	652-CR0402FX-47R0GLF	2	0.09	0.18	Bourns
R7-R9	652-CR0402FX-47R0GLF	3	0.09	0.27	Bourns
U1	511-STM32H735IGK6	1	13.50	13.5	STMicroelectronics

			Overall cost:	22.50	

### 4.3 Extension: USB-connectivity — BOM

**Table:** 

**Designator**	**Component**	**Number**	**Cost per unit EUR**	**Total cost EUR**	**Source of materials**
C1	187-CL05A105KO5NNNC	1	0.09	0.09	Samsung Electro-Mechanics
C2	187-CL05A106MP6NUN8	1	0.11	0.11	Samsung Electro-Mechanics
C3-C28	81-GRM033Z71C104KE4J	26	0.09	2.34	Murata
D1	750-CDBQR40	1	0.24	0.24	Comchip
EXT1-EXT2	571-1MMRD25VS00FTBP	2	7.33	14.66	TE Connectivity / AMP
EXT3-EXT4	571-1MMRD25VS00FTBP	2	7.33	14.66	TE Connectivity / AMP
IC1	511-LD57100J120R	1	0.48	0.48	STMicroelectronics
IC2-IC4	595-TPS2052BDRBR	3	0.97	2.91	Texas Instruments
IC5	579-USB5807C/KD	1	8.12	8.12	Microchip
J1-J3	640-USB4105-GF-A-060	6	0.74	4.44	GCT
R1	RC0402DR-0712KL	1	0.10	0.1	Yageo
R2-R4, R19	652-CR0402FX-2003GLF	4	0.09	0.36	Bourns
R5-R6, R20, R22, R25-R26	652-CR0402FX-4701GLF	6	0.09	0.54	Bourns
R7-R18	603-AF0402FR-0756KL	12	0.09	1.08	Yageo
R27	652-CR0402FX-4701GLF	1	0.09	0.09	Bourns1
Y1	520-1612MV-250-CNT	1	1.46	1.46	ECS International

			Overall cost:	51.60	

Note on the USB extension BOM: the J1-J3 components contain two USB-C ports, one on the top side and one on the bottom side of the PCB. This makes a total of six USB-C USB4105-GF-A-060 ports.

### 4.4 Extension: Serial-connectivity — BOM

**Table:** 

**Designator**	**Component**	**Number**	**Cost per unit EUR**	**Total cost EUR**	**Source of materials**
C1-C6	80-C0402C104K4R7867	6	0.11	0.66	KEMET
EXT1-EXT2	571-1MMRD25VS00FTBP	2	7.33	14.66	TE Connectivity / AMP
EXT3-EXT4	571-1MMRD25VS00FTBP	2	7.33	14.66	TE Connectivity / AMP
IC1	595-TRSF3221EIRGTR	1	2.13	2.13	Texas Instruments
J1-J2	538-22-05-7045	2	0.19	0.38	Molex
J3	437-8501000420001101	1	1.31	1.31	Preci-Dip
Q3	621-DMN1260UFA-7B	1	0.29	0.29	Diodes Inc.
R1, R4	603-AC0402FR-07120RL	2	0.09	0.18	Yageo
R2-R3	652-CR0402AFX1002GAS	2	0.09	0.18	Bourns
R5-R7	652-CR0402FX-4701GLF	3	0.09	0.27	Bourns
U1	700-MAX3485AEASA+	1	5.74	5.74	Analog Devices

			Overall cost:	40.47	

## 5 Build instructions

### 5.1 Potential safety hazards

When soldering, wiring, cutting or drilling, please take all necessary safety precautions.

### 5.2 Design choices

A fundamental design decision was to make the main board as small as possible and to outsource the necessary functionalities to extra boards. This approach has the advantage of significantly reducing the necessary space, while also promoting modularity. All logical components have been selected or designed as modules. In particular, this applies to the tty2eth module, the SoM, the PoE module, and the extension boards. Despite the intricate and diminutive nature of the Surface Mount Devices (SMD) chip selection, the modularity allows for relatively uncomplicated repairs by replacing the defective modules.

### 5.3 Build the Gateway-Module-v3

#### 5.3.1 Manufacturing the PCBs

The Gateway-Module-v3 is constructed with several key components, including the PCBs of the individual boards, the SoM phycore-imx8mp from the company Phytec [18], and the appropriate heat sink. The latter component can be procured from a Computer Numerical Control (CNC) manufacturer by providing the 3D step file. In this project, copper was selected for its thermal characteristics.

The fabrication of these PCBs must be conducted by a specialized PCB manufacturer. In the context of high-speed signals, the appropriate impedances play a significant role. The design of these boards was developed in accordance with the capabilities and impedance specifications from JLCPCB [33].

However, it is possible to utilize a different manufacturer, as modifications may be necessary to ensure compatibility with different capabilities and impedance characteristics. The relevant Gerber files, along with their respective impedance specifications for JLCPCB, are listed in Table 2.

**Table 2:** List of all Gerber files and their associated controlled impedances.

Gerber filename	Controlled impedance	Location of the file
usb-extension_1.zip	JLC06161H-3313	pcbs/production/usb-extension_1.zip
tty2eth-nano_1.zip	JLC04161H-7628	pcbs/production/tty2eth-nano_1.zip
serial-extension_2.zip	JLC04161H-7628	pcbs/production/serial-extension_2.zip
gwm-main-board_1.zip	JLC08161H-2116	pcbs/production/gwm-main-board_1.zip

#### 5.3.2 Soldering the components

In order to successfully solder components to circuit boards, it is strongly recommended to utilize a soldering iron, a heat plate, and a heat gun. As an alternative solution, it is conceivable to utilize an assembly service for the assembly of the boards. In this work, manual soldering was performed. It is generally recommended that large and challenging SMD be soldered first with the heat plate or the heat gun, and that particular caution be exercised with the plastic part, i order to prevent the occurrence of melting damage to the components.

**Fig. 4:**
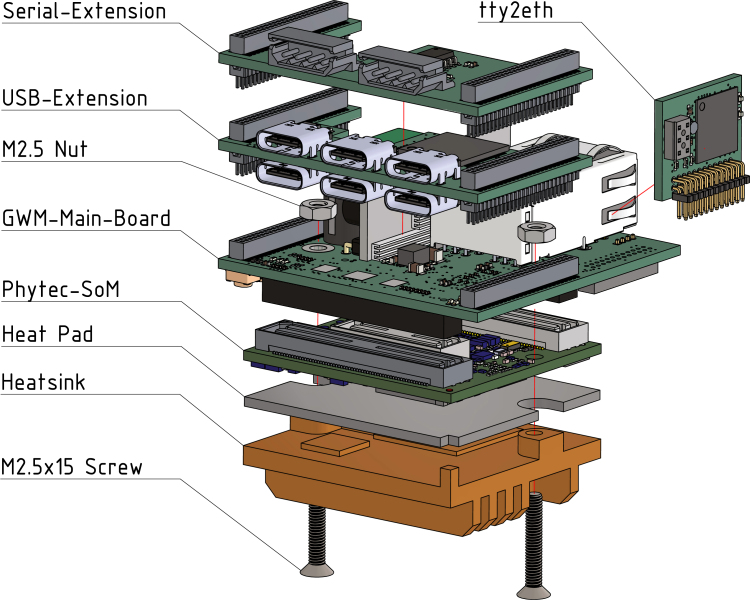
Gateway-Module-v3 assembly.

The interactive BOM can be utilized during the soldering process to monitor progress and provide a visual representation of the components orientation and location on the boards. Upon completion of all PCBs, the Gateway-Module-v3 must be assembled in accordance with the illustrated instructions in Fig. 4.

#### 5.3.3 Installing

Once the Gateway-Module-v3 has been assembled, the software can be installed and a first functionality test can be performed. In order to successfully complete the installation of the firmware and the operating system, it is essential that the Gateway-Module-v3 is supplied with a power source. This can be accomplished by utilizing either the PoE Ethernet port or the USB-C port that is located on the front of the device. When the system is powered on, the orange LED on the tty2eth module should be illuminated.

##### Firmware of tty2eth:.

**Fig. 5:**
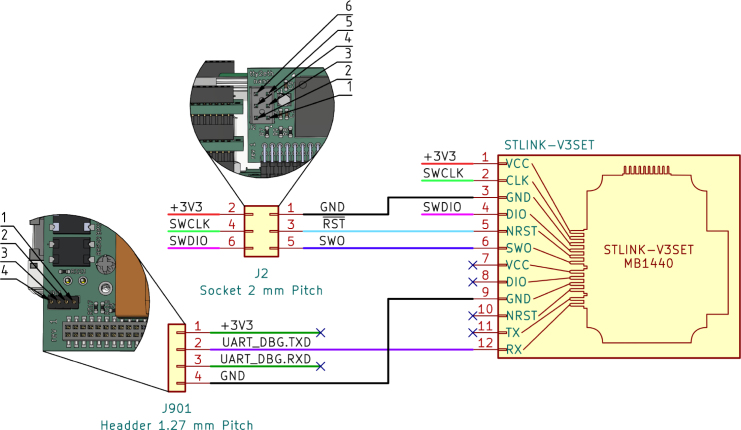
STLINK-V3 SWIO and UART connection diagram.

The tty2eth module requires its own firmware. A prebuilt version of the firmware can be found in this project, and the source code with the build instructions can be downloaded at [30]. In order to perform a firmware update on the tty2eth module, it is necessary to utilize a STM32 flush device, such as the STLINK-V3SET [34], along with a connection cable. In addition to receiving debug messages from the tty2eth module, the STLINK-V3SET can be utilized as a UART reader. To do so, it must be connected to the J901 connector on the main board of the Gateway-Module-v3. In this case, small clamps should be sufficient; alternatively, a 4-pin Molex PicoBlade connector could be used. For both connections, Fig. 5 can be used as a reference.

Run the following commands from Listing 1 to flush the prebuild firmware form the current directory. This process needs the OpenOCD application.

**Figure:**
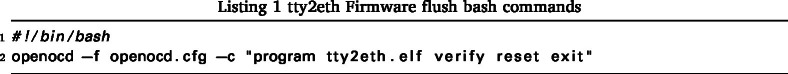

Following the successful flushing of the firmware, the boot log over the UART connection of the tty2eth module should display the Internet Protocol (IP) address of the device. By default, the module utilizes Dynamic Host Configuration Protocol (DHCP) for IP configuration. The configuration of the tty2eth module can be obtained and modified via SSH File Transfer Protocol (SFTP). It is strongly recommended to use of SSH keys or strong passwords to enhance the security of the usage of this module. Further information regarding usage and features can be found in Section 6.

##### Linux on Gateway-Module-v3 :.

**Fig. 6:**
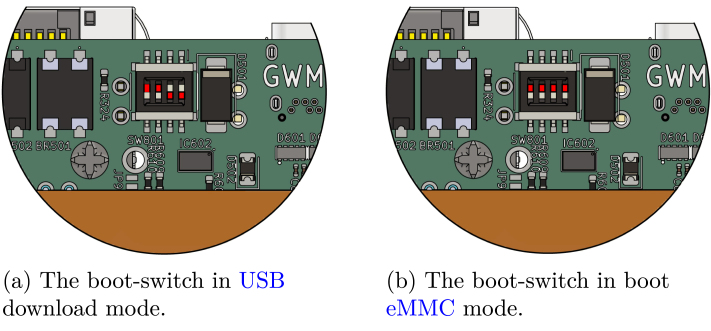
The location of the boot-switch on the Gateway-Module-v3.

The SoM of the Gateway-Module-v3 runs an ARM-based Linux operating system. This Linux distribution is based on the mkbox project [31], [35] and is based on a minimal Alpine Linux designed to create application-specific images. To install the desired system on the Gateway-Module-v3, the boot mode must be set to Serial Download over USB mode. This is done by setting the boot switches (SW801) (Fig. 6) on the Gateway-Module-v3 to 0011, as shown in Fig. 6(a). This configuration enables writing to the eMMC memory via the USB interface on the front side. Once the boot configuration has been made, the Gateway-Module-v3 can be connected to the PC. If the power supply from the USB C port is insufficient, additional power can be supplied via the PoE connection. Then, the Linux image can be installed on the Gateway-Module-v3 using the command from Listing 2. The image is unpacked with ztdcat and installed on the eMMC memory using uuu and the U-Boot bootloader flash.bin file. Updates or changes to the operating system can be carried out in the same way. If the mkbox-project is used, the automatic update mechanism can be used as an alternative [31]. To boot Linux from the eMMC, set the boot switch to 0000 (see Fig. 6(b)). After restarting, the boot process should be visible in the tty2eth module.

**Figure:**
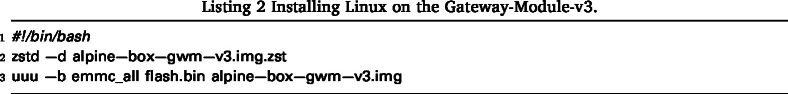

**Fig. 7:**
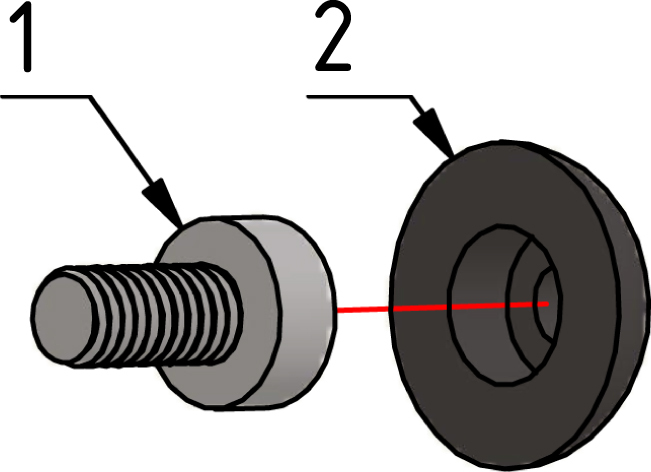
Glue the M2 × 5 screw (1) in the feet part (2). Repeat this step 3 more times.

### 5.4 3D printing — case

The project includes a 3D-printed case that serves as an enclosure for the device. The necessary parts and printing material can be found in Table 3. It should be noted that modifications to the backplate can be made in order to suit the selected extension boards. In this scenario, a USB extension and a serial extension board are utilized. In the absence of a 3D printer, 3D printer services are available online. In this work, the case was printed with carbon reinforced PLA, and a Bambu Lab X1C was utilized. In order to optimize heat resistance and stability, the utilization of a nylon material is recommended. The prices for the 3D printed parts in Table 1, Section 4 are based on nylon as the print material and using a 3D printing service.

**Table 3:** List of all 3D printing files and their recommended printing materials.

Step filename	Material	Location of the file
case-base.step	PLA or Nylon	3d-case/case-base.step
foot.step	PLA or Nylon	3d-case/foot.step
fan-grill.step	PLA or Nylon	3d-case/fan-grill.step
backplate.step	PLA or Nylon	3d-case/backplate.step

### 5.5 Build all together

As described in Section 4, Table 1 lists the requisite components for the assembly of the Gateway-Module-v3.

#### Prepare feet:.

First, four M2 × 5 screws need to glue or press in the foot pieces, as described in Fig. 7.

#### Prepare fan:.

**Fig. 8:**
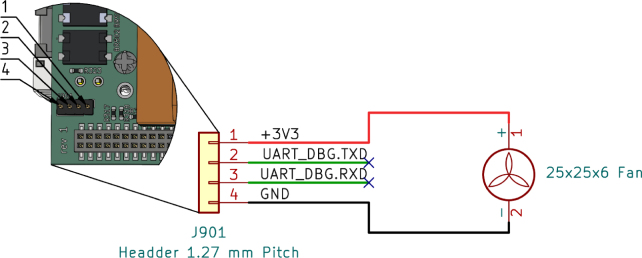
Fan cable pinout and connector references.

To prepare the 25 × 25 × 6 fan, crimp the fan wire to a 4-pin Molex PicoBlade 1.25 mm connector, see Fig. 8 for reference.

#### Prepare screen:.

**Fig. 9:**
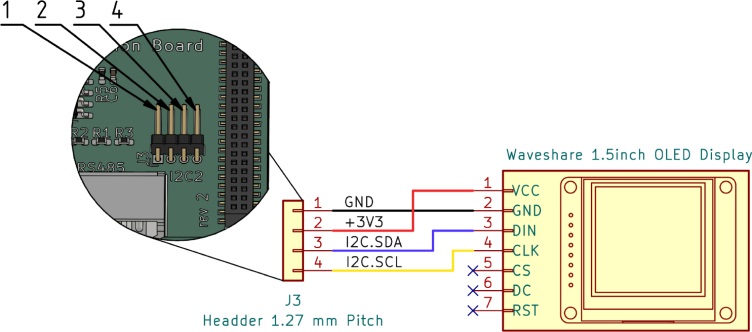
Screen cable pinout and connector references.

The supplied cable and connector of the OLED display can be used, but the Gateway-Module-v3 side of the cable needs to be modified and a 4-pin Molex PicoBlade 1.25 mm connector should be used instead of the larger single-pin connectors. Fig. 9 shows the exact wiring diagram.

#### Prepare serial cables:.

**Fig. 10:**
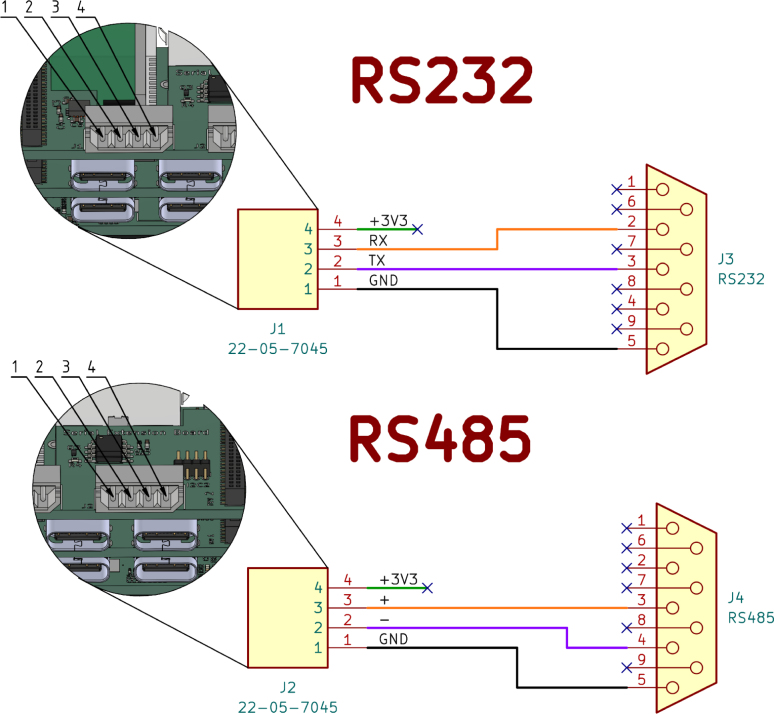
RS-232 and RS-485 cable pinout and connector references.

The Gateway-Module-V3 is too small for conventional RS-232 and RS-485 connectors, two Molex 22-05-7045 connector are used instead. See Fig. 10 for a reference on crimping the correct RS-232 and RS-485 cables.

#### Step 1:.

**Fig. 11:**
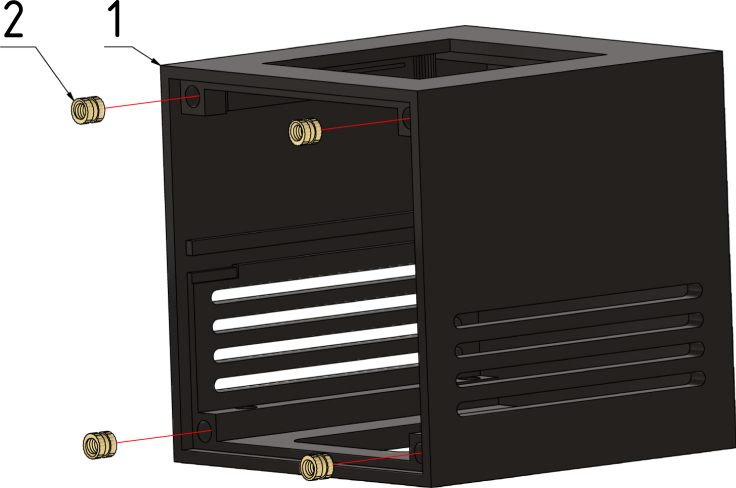
Step 1, base of the case (1) and M2 × 4 × 3.2 threaded inserts (2).

The assembly of the case starts with the case base (1) and the four threaded inserts M2 × 4 × 3.2 (2) are pressed into the front side of the case with a soldering iron until their front side is at the same level as the front side of the case, as shown in Fig. 11. Be careful with the hot soldering iron and the hot threaded inserts.

#### Step 2:.

**Fig. 12:**
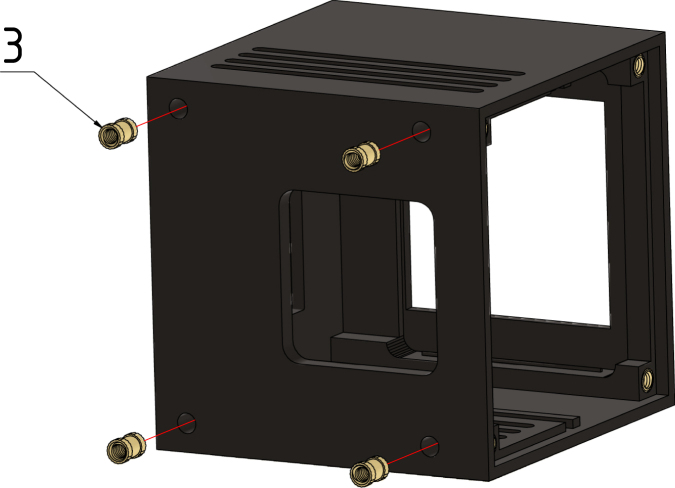
Step 2, M2 × 4 × 3.2 threaded inserts (3).

Repeat the previous step with four M2 × 4 × 3.2 threaded inserts (3) on the bottom of the case, see Fig. 12.

#### Step 3:.

**Fig. 13:**
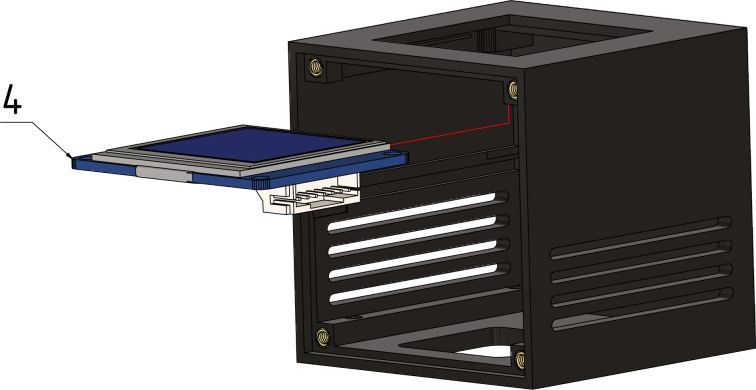
Step 3, OLED Screen (4).

Press the OLED display (4) from the inside to the top of the case (Fig. 13) and connect the wire to the J3 connector on the Serial-Extension board, see Fig. 9 for reference.

#### Step 4:.

**Fig. 14:**
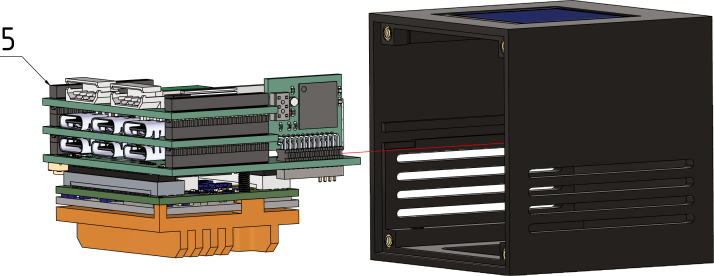
Step 4, Gateway-Module-V3 (5).

In preparation for step 5, connect the fan wire through the hole on the bottom side to the J901 port of the main board, see Fig. 8 for reference. Next, slide the Gateway-Module-V3 (5) into the center inlet of the case. Make sure it fits well (see Fig. 14).

#### Step 5:.

**Fig. 15:**
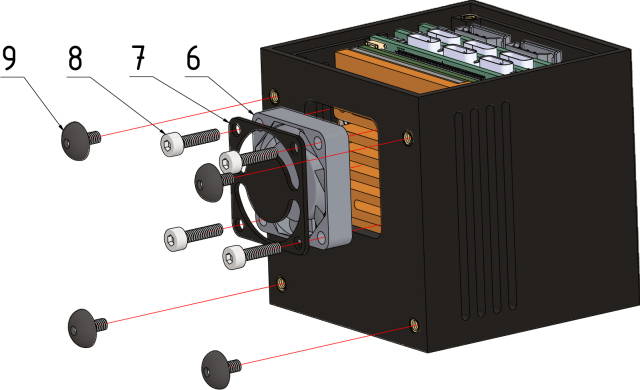
Step 5, 25 × 25 × 6 fan (6), fan grill (7), M2.5 × 10 screws (8) and the case feets (9).

Place the 25 × 25 × 6 fan (6), its wires – be careful not to damage the wires – and the fan grill (7) on the bottom of the case. Screw all parts together with M2.5 × 10 screws (8) to the heat sink of the Gateway-Module-v3. Next screw the feet (9) from the preparation step to the bottom of the case, see Fig. 15.

#### Step 6:.

**Fig. 16:**
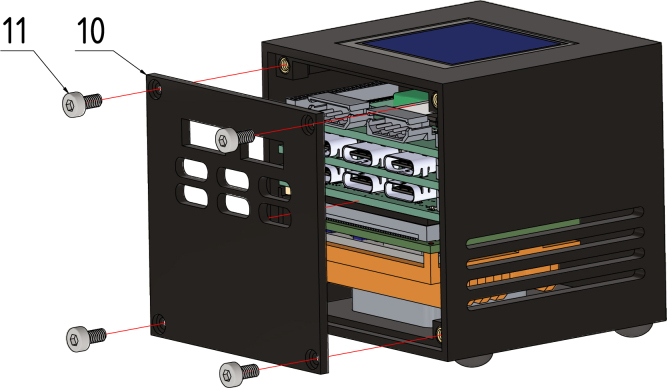
Step 6, backplate (10) and M2.5 × 5 Screws (11).

Finally, screw the case backplate (10) to the case back using four M2 × 5 screws (11) (see Fig. 16).

**Fig. 17:**
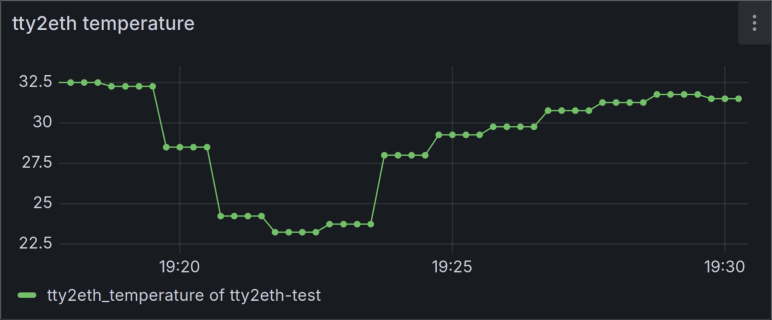
Graphana Dashbord of the Temperature sensor from the Gateway-Module-v3.

## 6 Operation instructions

This section provides insight into operating the new Gateway-Module-v3 and the tty2eth module. After a successful installation, the following functions and features will be available:

### 6.1 Management module tty2eth

The tty2eth management module provides users with three interfaces for interaction: SSH for direct hardware control, an HTTP/HTTPS server for Prometheus metric export, and SFTP for downloading and uploading the configuration.

#### 6.1.1 SSH connection

After starting the Gateway-Module-v3, the tty2eth module boots up and establishes a network connection. It then provides an SSH connection on port 22. The default IP configuration setting is DHCP, which can be changed in the settings (see Section 6.1.3). After successfully logging in to the SSH connection (the default username is “root” and no password is needed), a menu appears that provides basic functionality (see Listing 3).

**Figure:**
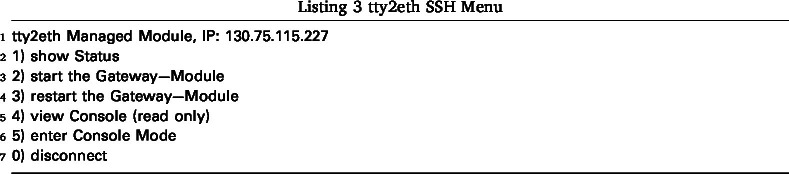

From the main menu, press 1 to view the current status of the Gateway-Module-v3, as shown in Listing 4. Depending on the power status of the Gateway-Module-v3, press 2 or 3 to start, stop, or restart the main CPU phycore-imx8mp. The remote console can be started in two modes: read-only mode with 4 or shell mode with 5. Both modes show the debug serial console of the main CPU of the phycore-imx8mp. Through this console, users have shell access to the Linux system. One advantage over a normal SSH connection is that this console also displays the boot process and provides access to the bootloader if needed. The main difference between read-only mode (View Console) and shell mode (Enter Console Mode) is that the user can send commands in shell mode. Once shell mode is activated, you are connected to the serial console. In the standard Linux configuration, you are logged in as root and can send commands. To exit read-only mode, press any key. This will display the main menu again. Shell mode can only be exited by ending the SSH session. The SSH users of the tty2eth module are separate from the users of the Gateway-Module-v3 and must be managed separately. The Linux console can be configured to prompt for a login when it starts. Note that tty2eth performs a one-to-one translation of the serial console from the phycore-imx8mp processor to the tty2eth SSH session. All users in shell mode have the same shell and prompt. The connection can be closed and terminated by pressing the 0 key.

**Figure:**
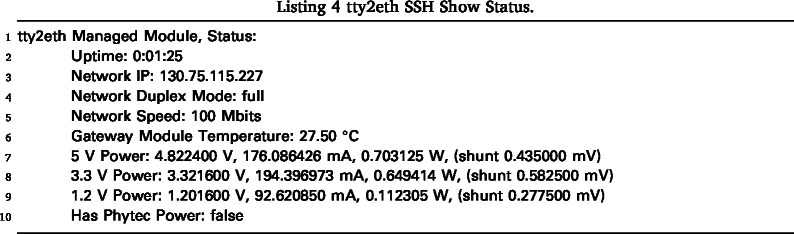

#### 6.1.2 Remote metrics

In addition to providing remote maintenance at the shell level, the tty2eth module can monitor the Gateway-Module-v3’s status and alert users in case of critical events. Which is possible by providing sensor values as Prometheus metrics. Monitoring can then be performed using Prometheus and Grafana. In this combination, Prometheus serves as a database for the sensor values, and Grafana visualizes and monitors the stored sensor values. The general setup of the Prometheus target can be found in [36], and the connection between Prometheus and Grafana is explained in [37], [38]. Once the sensor values are available in Grafana, dashboards with plots of the sensor values can be created. For example, see Fig. 17 with temperature values. In this figure, the temperature of the device was measured, and a temperature drop could be observed while an additional cooling system was running.

#### 6.1.3 Configuration

The tty2eth module configuration can be accessed via SFTP. The main configuration file (config.json) and the certificate files for the metric server (metric-server.crt and metric-server.key) are available. These files can be downloaded and uploaded again as described in Listing 5 and Listing 6.

**Figure:**
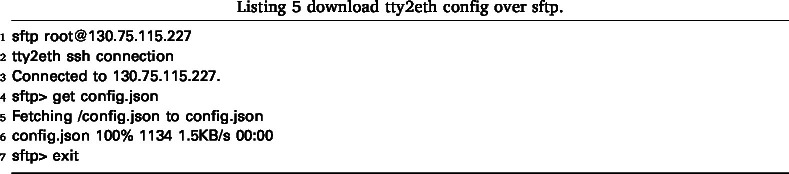

**Figure:**
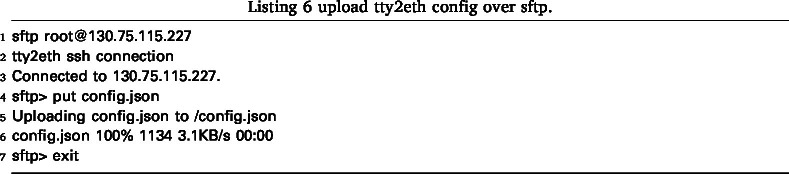

An example configuration is shown in Listing 7. The following settings can be configured: The unique SSH server host key can be stored in sshd.hostKey; if it does not exist, a new one will be generated when the system starts up for the first time. The users who have SSH and SFTP access are defined in users. Each user can be assigned a password or several SSH keys, which grant access to the SSH connection. The user password is hashed using the SHA-256 algorithm and stored in hexadecimal form. It can be generated as described in Listing 8. Alternatively, a user can be configured with no-auth. For this user, the password prompt is disabled, and the user always has access to the SSH connection. This is only recommended for the initial configuration.

The network can be configured in network either in DHCP mode or with a static IP address, for which the options network.ip, network.gateway and network.dns must be set.

**Figure:**
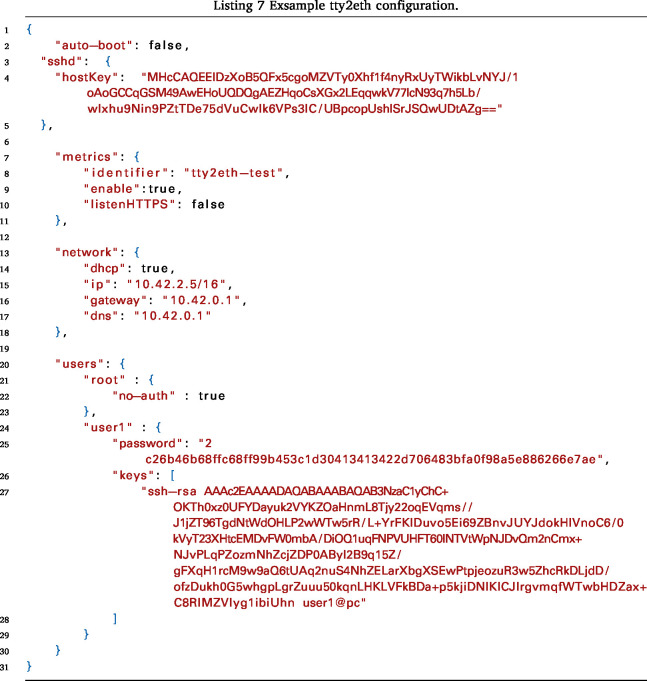

**Figure:**
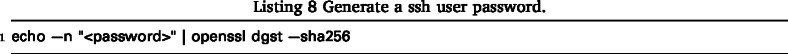

The Metric Server can be enabled or disabled using the setting metrics.enable. The Metric Server supports HTTP on port 80 or HTTPS on port 443. This setting can be configured with the metrics.listenHTTPS option. Once the server is configured to use HTTPS, a valid server certificate must be uploaded to the tty2eth module. This can be done using the destination files /metric-server.cet and /metric-server.key, in the same way as uploading the main config file in Listing 6. In addition, an identifier can be set in order to distinguish between the metrics of several devices. Once a setting has been changed, the tty2eth module must be restarted for the changes to take effect, except for user modifications.

**Fig. 18:**
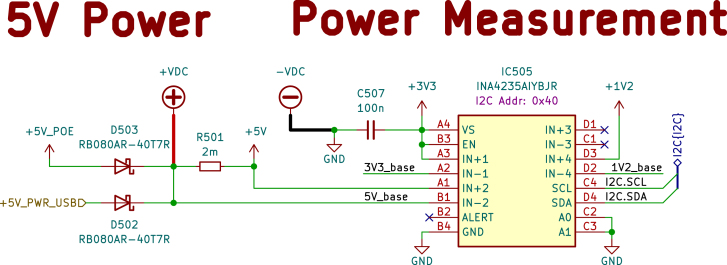
This figure is a simplified representation of the internal power measurement circuit. It also shows additional power injection points, +VDC and -VDC.

**Fig. 19:**
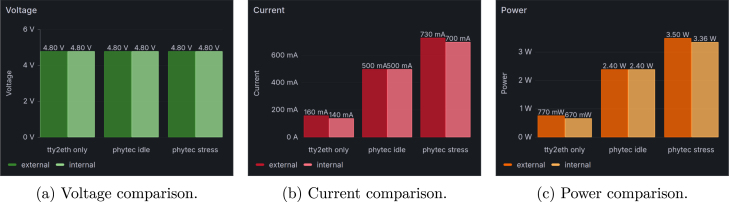
Results of internal and external measurements of the 5 V power line.

### 6.2 Gateway-module-v3 linux

The included operating system is based on the mkbox-project [31], [35] and consists of a minimal Alpine Linux. Each image is customized for a specific application. The Yocto project used by Phytec can also be used on the Gateway-Module-v3. In this case, the device tree must be adapted to the hardware of the Gateway-Module-v3. The device tree file imx8mp-gwm-v3.dtb inside the boards/gwm-v3/linux.patch from the mkbox project repository [32] can be used as a reference for this. After the operating system is installed and the boot switches are correctly set, see Section 5.3.3, the Gateway-Module-v3 can be started. The boot process can be monitored using the tty2eth module. Once the Gateway-Module-v3 has started, an SSH connection can be established. From this point on, all application options comparable to those of the SBC are available to the Gateway-Module-v3.

#### 6.2.1 Remote workflow

A proven method for working with Gateway-Module-v3 or other SBCs is to use VSCode [39] and the Remote SSH extension [40]. This allows users to access the familiar graphical environment of VSCode on remote Linux systems, even if no graphical user interface is installed. Users can edit files and manage projects directly on the Gateway-Module-v3 over the network and execute scripts and commands in the included terminal. A detailed Guild with installation and configuration instructions can be found in [41]. Strong passwords or, better yet, SSH keys are also recommended here. The latter, together with SSH-agent, offers the convenience of not having to enter the SSH password for every action. Setup on Linux or Windows systems may depend on the system and security requirements and may vary.

## 7 Validation and characterization

### 7.1 Internal power sensor testing

To ensure the accuracy of the internal voltage, current, and power measurements, they were compared with an external measurement. However, it is not possible to compare the incoming power currents from PoE or USB with the internal sensor values. Among other components, a PoE-to −5V voltage converter is installed before the internal 5 V measuring point, and an unknown amount of energy is lost at the prior components. Therefore, a 4.8 V voltage (the internal voltage level at the 5 V power lane) was injected with a laboratory power supply in front of the R501 shunt resistor of the 5 V power lane, as shown in Fig. 18. The test was carried out in three phases: once in standby mode with only the tty2eth module switched on, once with the phycore-imx8mp in idle mode, and once during a CPU stress test. Internal measurements were taken within a two-minute window, and the average value was calculated. The external measurements were read from the laboratory power supply. The results, which show a correlation between the two types of measurements, are shown in Fig. 19.

### 7.2 Benchmark

**Fig. 20:**
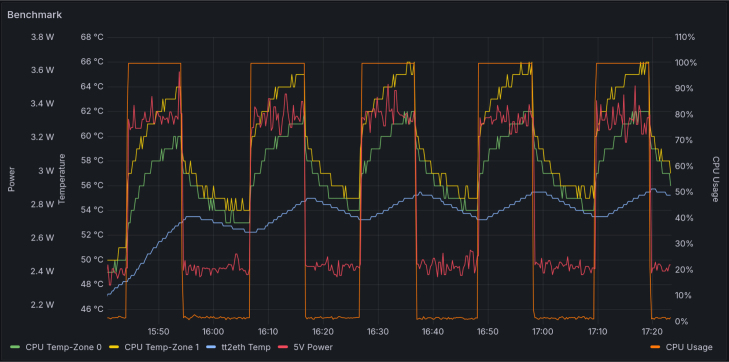
Shown are the system metrics of the five benchmark runs. Green shows the temperature of CPU Zone 0, yellow displays the temperature of CPU Zone 1, blue indicates the case temperature measured by the tty2eth module, red shows the power consumption of the 5 V power line, and orange indicates the single benchmark runs by CPU measurements.

In order to gauge performance and emulate recurrent loads on the system, a series of five 10 min benchmark runs were executed. Each benchmark was followed by a 10 min cooling phase. Throughout the entire process, the system was monitored, and the following metrics were recorded: temperatures of CPU Zones 0 and 1 and case temperature, measured with the tty2eth module; power consumption of the 5 V power line; and CPU usage, which served as an indicator of the individual benchmark runs. All CPU cores were loaded evenly using four CPU stress workers as stressors. The tool stress-ng was used as the benchmark. The benchmarks were evaluated using the bogo ops/s metric, which measures stress operations per second. The CPU execution time was used as the time basis. The measured metrics are shown in Fig. 20, and a summary of the metrics is shown in Table 4. As you can see, the performance remained stable over the five runs, with a value of 98.948bogoops/s. The maximum and initial temperatures remained similar, though a slight increase is evident. A similar power peak in the 5 V line was measured in all five runs.

**Table 4:** This table contains reduced metrics data from five benchmark runs.

Measurement	Mean	StdDev	Variance
performance [bogo ops/s]	98.9480	0.5032	0.2533
CPU max temp [°C]	65.4000	0.8000	0.6400
case max temp [°C]	54.8200	0.9453	0.8936
starting CPU temp [°C]	54.0000	1.5492	2.4000
starting case temp [°C]	52.1400	1.9653	3.8624
max power peak [W]	3.4960	0.0634	0.0040

### 7.3 Extensive stress testing

**Fig. 21:**
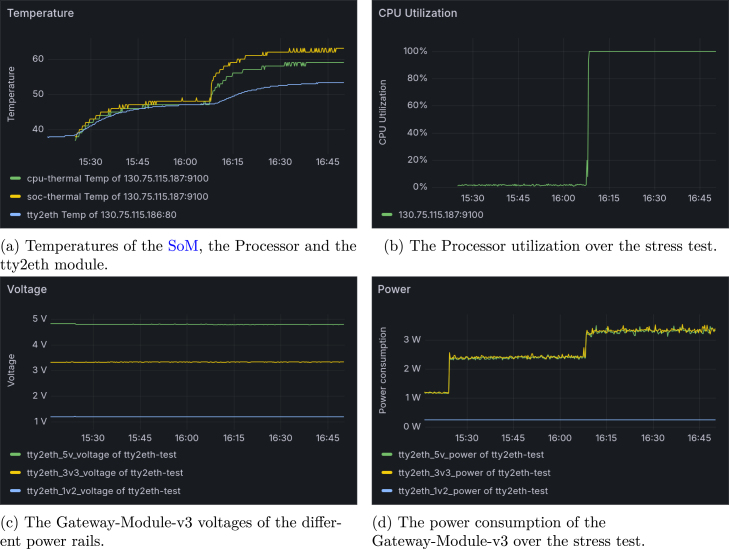
Grafana monitoring of the stress test.

To demonstrate the capabilities of the new Gateway-Module-v3, a 90 min stress test was conducted to push it to its limits in the laboratory. The test was divided into three phases: the start phase, during which only the tty2eth module ran; the idle phase, which occurred after the main system started; and the stress test, which used the stress-ng tool. During the test, all four CPU cores were utilized at 100%. Throughout the test, temperatures of various Gateway-Module-v3 sensors, power consumption, and voltage of the Gateway-Module-v3’s power rails were measured. The CPU utilization of the main processor was also measured. This measurement was taken using the tty2eth module and the SoM, and it was monitored in Grafana (see Fig. 21). After the initial start at 0 min (15:16), the entire Gateway-Module-v3 consumed 1.17W, and the tty2eth module temperature was 38°C. After the SoM started, the temperature rose and stabilized at around 46°C at 29 min (15:45), with the Gateway-Module-v3 consuming 2.4W. The CPU utilization increased to 100% at 52 min (16:08), with a maximum consumption of 3.4W. During this phase, the maximum temperatures were 53.25°C for the tty2eth, 63°C for the SoM, and 59°C for the processor. It should be noted that the SoM’s and the processor temperature increased significantly faster than that of the tty2eth module. This difference can be attributed to the design and the different positions of the temperature sensors. Throughout the entire test period, the Gateway-Module-v3 demonstrated a stable power supply, and no voltage changes were monitored. According to Phytec’s specifications, the phycore-imx8mp SoM can reach a maximum temperature of 85°C. This shows that the new Gateway-Module-v3 can be used under high loads in the laboratory. The entire test was carried out in a laboratory environment at a room temperature of 20°C.

### 7.4 Laboratory show case

**Fig. 22:**
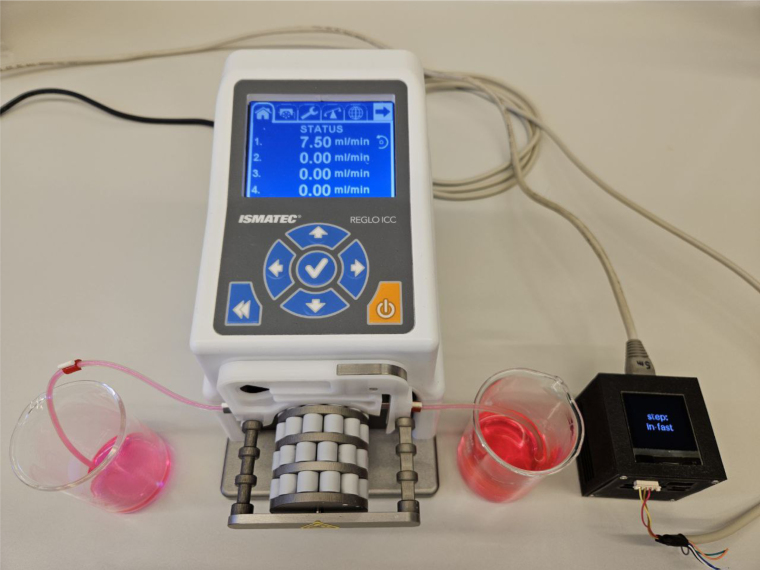
The experimental setup of the showcase. The Gateway-Module-v3 on the right, the Ismatec pump in the middle, and two glass-beakers next to the pump, filled with colored water.

The Gateway-Module-v3’s functionality was tested and demonstrated using a showcase. For this experiment, the RS232 connection on the serial extension board was used to control a Ismatec pump. The goal was to pump water from point A to point B at different flow rates. Additionally, the OLED display showed the status of the experiment. The entire setup is shown in Fig. 22. The Ismatec ISM4408 pump was connected to the RS232 extension board via a serial cable. The Gateway-Module-v3 was connected to the laboratory network via Ethernet so that it could be accessed from a user PC. The execution of this experiment involved the utilization of the mkbox image alpine-box-gwm-v3.img.zst from [29] . This image was installed as described in Listing 2. The connection can be tested, and the main CPU can be started from a PC using SSH, as described in Listing 9. The boot process can be monitored using View Console mode (4).

**Figure:**
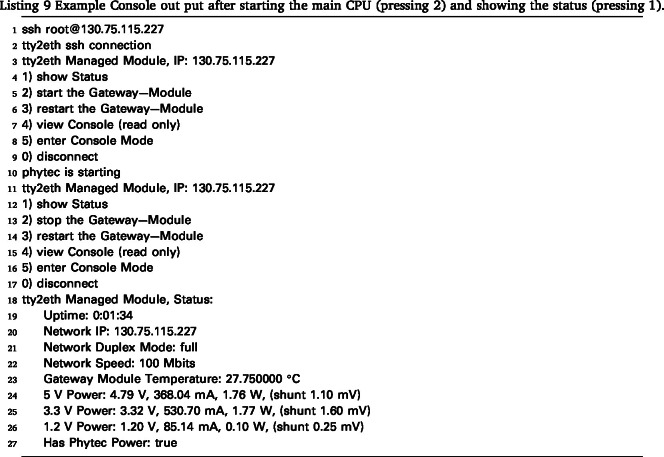

Once the Gateway-Module-v3 has booted completely, a remote SSH connection to the main system phycore-imx8mp can be established in VSCode. To accomplish this, the SSH-Remote extension must be installed in VSCode on the PC. References from Section 6.2.1 can be used as a guide. After establishing the connection, copy the Python script files from the demo folder in [29] to the Gateway-Module-v3. Then, the demo.py script can be executed. This script controls the pump, pumping the fluid from A to B and back again at different flow rates. The individual steps, including parameters and metadata, are recorded and saved in a JSON file according to the FAIR criteria. An excerpt from the file is shown in Listing 10. The file can then be loaded into a corresponding laboratory information management system (LIMS) or database for later evaluation or processing.

**Figure:**
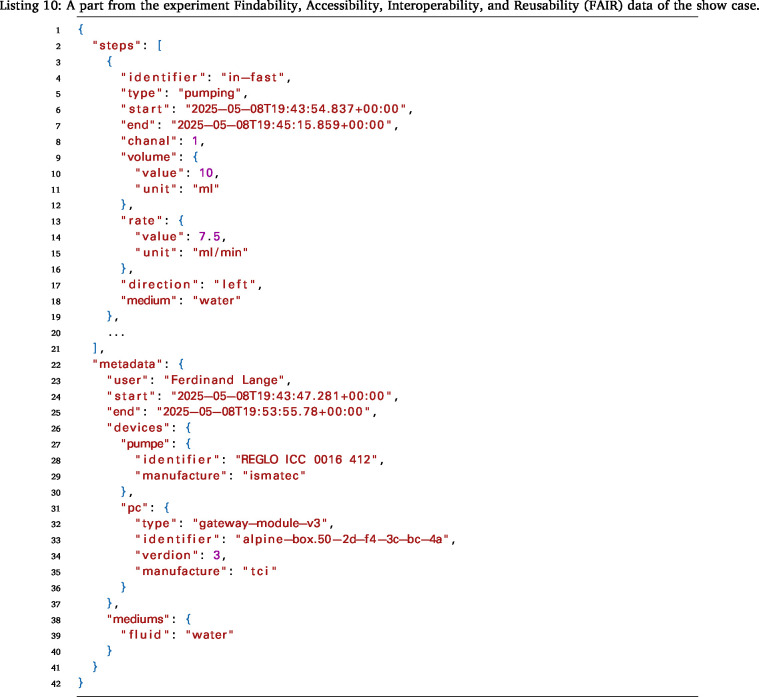

### 7.5 Capabilities and limitations

#### Capabilities:.

• Server like mini Computer, with modern hardware
• A compact design with integrated PoE support for reduced wiring
• Integrated remote maintenance and monitoring capabilities are offered.
• Device for laboratory digitalization
• Starting platform for self-build experiments
• Enables device control and data acquisition
• With support for application-specific Linux images (mkbox-project) [31]
• Modular design and versatile connection options

#### Limitations:.

• Advanced soldering skills or assembly services are required
• Higher prize than the original Gateway-Module

## 8 Conclusion

As laboratory equipment becomes increasingly digitized and customized, computer-controlled experiments are becoming more prominent in academic research environments. Consequently, the demand for SBC is increasing. For this purpose, this article introduces the new Gateway-Module-v3. The article describes the components of the new version and the methodology of their assembly. Furthermore, the necessity of remote maintenance is outlined, along with the introduction of the remote management module tty2eth, which is an integral component of the novel Gateway-Module-v3. This additional module enables users to monitor the hardware of digital laboratory devices and access and debug them in the event of malfunctions or during the development phase. Finally, a demonstration was conducted to illustrate the application of Gateway-Module-v3 in a laboratory setting and to present several potential applications. The hardware presented is not limited to laboratory use and can be utilized in a variety of other settings.

## CRediT authorship contribution statement

**Ferdinand Lange:** Writing – original draft, Validation, Software. **Sascha Beutel:** Writing – review & editing, Supervision, Project administration.

## Ethics statements

No humans subjects or animals were involved in the studies that lead to the publication of this article.

## Declaration of competing interest

The authors declare that they have no known competing financial interests or personal relationships that could have appeared to influence the work reported in this paper.
